# Dimensional Assessment of Psychopathy and Its Relationship with Physiological Responses to Empathic Images in Juvenile Offenders

**DOI:** 10.3389/fpsyt.2013.00147

**Published:** 2013-11-14

**Authors:** Daniel Martins de Barros, Alvaro Machado Dias, Antonio de Padua Serafim, Gustavo Bonini Castellana, Maria Fernanda Faria Achá, Geraldo F. Busatto

**Affiliations:** ^1^Institute and Department of Psychiatry, University of São Paulo, São Paulo, Brazil; ^2^Center for Interdisciplinary Research on Applied Neurosciences: NAPNA, São Paulo, Brazil; ^3^Clinical Neuroscience Laboratory, Federal University of São Paulo, São Paulo, Brazil; ^4^Methodist University of São Paulo, São Bernardo do Campo, São Paulo, Brazil

**Keywords:** psychopathy, youngsters, electrophysiology, IAPS

## Abstract

**Objective**: Many psychophysiological studies investigate whether psychopaths present low levels of electrodermal activity (EDA). However, despite evidence that varying degrees of psychopathy are normally distributed in the population, there is a paucity of EDA studies evaluating dimensionally. Moreover, although lack of empathy is a cornerstone of psychopathy, there has been a lack of studies using pictures of empathic emotional content to assess psychophysiological responses.

**Method**: We studied a population of young male delinquents (*n* = 30) from a detention center, using the Psychopathy Checklist Revised (PCL-R) to determine if higher levels of psychopathy were related to lesser degrees of EDA in response to emotion-eliciting pictures of empathic content.

**Results**: There were significant correlations (*p* < 0.05) between latency and peak of EDA responses to unpleasant pictures and factor 1 scores, as well as between lability of EDA responses and factor 2 scores.

**Conclusion**: These results extend previous findings indicating direct relationship between EDA and psychopathy, and suggest that separate investigations of the two PCL-R factors have the potential to unravel more complex relationships between EDA and psychopathy. Also, by demonstrating such associations using emotion-provoking stimuli with empathic content, our results provide a link between levels of psychopathy and biological indices of empathic detachment.

## Introduction

Since the first conceptualization of psychopathy by Kurt Schneider as a disorder in which the individual repeatedly causes suffering to others, “lacking in compassion, shame, honor, remorse and conscience” (1), the definition of this condition underwent profound variations. Notably, the initial view of psychopathy as a categorical diagnosis was challenged when Cleckley introduced the concept of core personality dimensions in non-psychotic offenders (2), arguing that constitutive factors of the personality could be, to a greater or lesser degree, associated with criminal behavior in some individuals, whom he labeled psychopaths. More recently, the systematization proposed by Hare et al. (3) further emphasized the notion of psychopathy as a quantifiable dimension normally distributed in the general population (4, 5). Psychopathy has since been predominantly delineated with basis on continuous psychometric scores, and research studies in this field flourished (3, 5).

The greatest contribution of Hare to systematic research on psychopathy consisted in the development of the Psychopathy Checklist (PCL) and subsequently its revised form, the Psychopathy Checklist Revised (PCL-R) (6, 7). This scale encompasses the two following factors, which in combination allow the establishment of the degree of psychopathy in a given individual: “factor 1,” which establishes whether the subject presents traces of coldness, absence of regret, cruelty, insincerity, and lack of empathy (the latter of which is seen as a central feature of psychopathy); and “factor 2,” which evaluates the subject in relation to potential difficulties of self-control and criminal fickleness, as well as his antisocial behavioral repertory (5, 8, 9). Individuals with high scores on factor 1 can be seen as primary psychopaths, displaying a predominantly inborn core of coldness, shallowness, and non-empathy (4, 10), while subjects with high scores on factor 2 are regarded as secondary psychopaths, with a more environmentally influenced pattern of criminal behavior, and not so insensitive as those classified as primary psychopaths (10–12).

Psychopathic traits as assessed with the PCL-R have been shown to be normally distributed both in the general population and in criminals, and notably, criminal recidivism is significantly associated with higher PCL-R scores (13). Also importantly, PCL-R scores display a close relation with higher rates of violent behavior even for non-psychopaths (3).

In the last few years, several neurobiological studies have attempted to investigate whether subjects who receive a categorical diagnosis of psychopathy present low levels of autonomic arousal, reflecting their core callousness (14, 15). However, in only a small number of electrodermal activity (EDA) studies psychopathy was assessed using the PCL-R, with scores treated as continuous, dimensional variables (16).

Moreover, previous psychophysiological studies of psychopathy have used variable types of negative valence stimuli (aversive, anger-provoking, or painful), but none of such investigations have employed stimuli expected to produce empathic reactions (despite the fact that lack of empathy is a cornerstone in the evaluation of psychopathy). In a meta-analysis involving 95 studies, Lorber (17) demonstrated the existence of a significant yet modest association between the diagnosis of psychopathy and low EDA, as indicated by findings of hypo-reactivity to stimuli of negative emotional valence; still, he concluded that results were quite variable and often contradictory (17). In fact, despite an overall tendency to relate antisocial behavior with under-arousal (17, 18), a number of studies conversely show that anger-induced aggression and antisocial behavior may be facilitated by increased arousal (19–22).

Further psychophysiological studies using PCL-R scores as continuous variables are therefore needed, in order to investigate whether there are specific patterns of relationship between EDA measures and the two separate factors of the PCL-R.

In the present study, EDA measures were obtained in a population of incarcerated youngsters from a single institution, and correlated with scores on both factors 1 and 2 of the PCL-R in a dimensional fashion, as well as with criminal recidivism. EDA indices were obtained during presentation of images of variable emotion-provoking potential (pleasant, neutral, or unpleasant) of empathic content. We hypothesized that there would be significant negative correlations between higher PCL-R scores and the degree of callousness as shown by impaired autonomic reactivity during presentation of unpleasant pictures. We also predicted that crime recidivists would have higher PCL-R scores and lower levels of autonomic reactivity. Finally, we wished to investigate, on an exploratory basis, whether significant associations would be present not only for unpleasant stimuli but also for neutral or pleasant pictures.

## Materials and Methods

This study included juvenile offenders facing time-limited, “social-educative” sentences at the “Fundação Casa,” a correctional institution for minors convicted of serious crimes, in the state of São Paulo, Brazil. The facilities of Fundação Casa serve the whole state of São Paulo (where 3,200,000 15–21 youngster dwell) and harbor about 7000 minors, in several different blocks of buildings. This incarcerated population is typically composed of youngsters of low socioeconomic status, coming from single-parent homes and a high criminality environment (23). The specific block where this study was conducted consists of 5 buildings, where about 200 inmates are distributed according to their criminal profiles (e.g., non-recidivists versus recidivists).

Brazil’s law system defines that there is no minimum time but solely a maximum incarceration period that an underage offender can face, which is three consecutive years. Moreover, the incarceration period in this institution ceases at the completion of the 21st birthday. According to a recent survey that was held by the institution, around 25% of the inmates are older than 18 and younger than 21 (23). Recidivists are those individuals that have been incarcerated more than once, for different types of rule-breaking behavior.

Based on previous studies of our group (24), we predicted that the standard deviation (SD) of PCL-R scores for the incarcerated population would be nine points. Assuming a confidence level of 95% and a maximum error of three points, we estimated that it would be necessary to include at last 30 inmates in order to afford sufficient power to assure that our PCL-R scores reflected the profile of the whole population of incarcerated youngsters. Youngsters were eligible to this study if: aged 18–21; enrolled in, or having completed high school; and not receiving any psychopharmacologic agents.

### Clinical evaluation

With the aim of excluding the presence of psychiatric disorders from the sample, all eligible subjects underwent interviewing with the Brazilian version of the Mini-International Neuropsychiatric Interview (MINI) (24–26), a structured questionnaire of quick application (15–30 min) developed to fulfill criteria for major psychiatric disorders according both to DSM-IV and ICD-10.

### Psychopathy evaluation

The Brazilian version of the PCL-R scale (9) was applied with the aim of estimating the inmates’ levels of psychopathy. All interviews were applied by the same physician, who was blind to the results of the other tests that were undertaken by the participants.

This scale is applied as a semi-structured interview consisting of items that investigate the degree of psychopathic personality traits. It has 20 items, whose individual scores range from 0 to 2 points, so that the maximum score is 40 points. The cutoff for the presence of psychopathic traits has been established by Hare and collaborators as 15 points, whereas full blown psychopathy applies when scores are greater than 29 points (3).

In accordance with the PCL-R instructions in regard to young populations (27), three evaluative dimensions were excluded from the scoring criteria: (1) high level of short-term matrimonial relations; (2) promiscuous behavior; (3) criminal versatility. Final results were corrected for the exclusion of such dimensions.

### Psychophysiological evaluation

Electrophysiological data consisted of EDA measurements collected with a six-channel J&J I-330 oscilloscope (J&J Engineering, Poulsbo, WA, USA), from which two channels were used. The digital signal processing software BioExplorer 1.5 (CyberEvolution) was used to run the electrophysiological design, which presented Flash images to the subjects and collected physiological responses. The software was installed in a Sony Vaio notebook (2 GHZ, 15.4 screen), while the participants interacted with a second LCD monitor (19 inches), which presented the images.

The EDA measures were recorded from the second and third fingers of the non-dominant hand (MC-6SY cable), and the range values were established between 0 and 10 Ω. Such electrodermal responses were measured in relation to three parameters of interest: latency (time between stimulus presentation and peak amplitude measured in milliseconds); peak activation, defined as the amplitude of the electrophysiological curve, from baseline (the moment preceding the appearance of each stimuli) to the moment of higher activation during stimuli presentation (measured in microohm); and response lability (the number of micropeaks that occur in the overall response period between stimulus presentation and recovery to half-baseline, measured in microohm per second). We developed a macro that automated the process of selecting and exporting EDA measures to a template, from which the data from each individual subject regarding the three variables of interest were finally extracted (.xlx).

The core of our experimental design consisted in the recording of physiological responses under exposure to images selected from the International Affective Picture System (IAPS) (28), including pictures of unpleasant or pleasant emotional content, and pictures of neutral emotional content. The categorization of images in relation to these emotion-provoking dimensions was defined with basis on previous large-scale validation experiments (15, 29, 30). The use of IAPS pictures to induce emotional states has been previously validated for the Brazilian population (31), and one previous study from our group has reported the successful use of such picture inventory to assess psychophysiological indices in incarcerated individuals (adult prisoners), based upon the distinction between normal and abnormal reactions to images (24).

The experimental design included the presentation of three blocks containing nine images each, respectively consisting of three pleasant, three neutral, and three unpleasant pictures. The emotion-provoking images were selected according to their empathic content, privileging the depiction of two or more people engaged in situations involving positive or negative emotions, requiring the ability to understand and share other person’s feelings, such as scenes showing people in happy situations (e.g., a father carrying his smiling baby) or suffering with each other (e.g., a senior man besides his dying wife). The neutral images depicted plain objects (e.g., an umbrella, a book, or a wall clock). Each image appeared during 6 s and was followed by a 6-s blank screen.

### Statistical analysis

Comparisons of PCL-R scores between criminal recidivist and non-recidivist individuals was carried out using two tests: the total score was compared using Student’s *t* test, and factors 1 and 2 were compared using Mann–Whitney tests. Spearman tests were used to correlate PCL-R scores with the three EDA indexes: latency, peak response, and lability, for each type of image (unpleasant, neutral, and pleasant pictures). The significance level for all tests was set at *p* < 0.05.

## Results

During the 10-month period of data collection, we identified a total of 48 individuals who met the inclusion criteria for the study. Of this total, seven subjects were released from the facility between the psychophysiological assessment and PCL-R testing thus preventing their complete evaluation, and three were excluded due to psychiatric conditions as assessed with the MINI (one due to the presence of symptoms of obsessive-compulsive disorder, one due to depressive symptoms, and one due to panic attacks). Of the 38 remaining individuals, 1 refused to take part in the study and 7 were excluded due to corrupted EDA data.

All participants of the final sample (*n* = 30) were aged 18, with the exception of one inmate aged 17 and one aged 19 years (mean age = 18.0 years). The incarceration period of the cohort ranged from 3 to 17 months (mean incarceration period = 7.39 months). Five participants were incarcerated for homicide, only one of whom was a recidivist. Twenty-three individuals were incarcerated for robbery or theft, and the remainder for a variety of other offenses including drug dealing and illegal gun possession. Eleven subjects met criteria for marijuana dependence and 4 for cocaine abuse, with all subjects having abstained from drugs for more than 6 months.

No differences in age or incarceration time was identified between the final sample of individuals included in the study (*n* = 30) and subjects who initially met inclusion criteria but were excluded due to incomplete assessments (*n* = 18).

### Psychopathy checklist revised

Table 1 summarizes the PCL-R results, grouped by their scores on factors 1 and 2. A significant difference in psychopathy scores was found between the recidivist and non-recidivist populations for both factors (Table 1). The lower-than-estimated SD of PCL-R scores increased the power of the sample (0.97).

**Table 1:** **Scores on the PCL-R for recidivist and non-recidivist subjects**.

	*N*	Group	*M*	SD	Significance
Factor 1	17	Recidivists	4.24	2.19	*p* = 0.0087
	13	Non-recidivists	2.00	1.87	
Factor 2	17	Recidivists	12.24	3.88	*p* = 0.0028
	13	Non-recidivists	7.62	3.15	
Total score	17	Non-recidivists	18.74	5.40	*p* = 0.0006
	13	Recidivists	11.04	5.30	

None of the subjects scored above 23 on the PCL-R, thus indicating that no individual from the sample met formal criteria for psychopathy based on the recommended 30-point diagnostic threshold (27).

### Correlations between EDA measures and scores on the PCL-R

Table 2 shows the rho indices for the correlations of the three EDA parameters measured during presentation of unpleasant pictures versus PCL-R scores. Total psychopathy scores showed no significant correlations with EDA, but when analyzed independently, factors 1 and 2 revealed significant correlations. Positive correlations were found for factor 1 (denoting affective aspects of psychopathy including indifference and callousness): higher scores on this parameter were associated with longer physiological response times to unpleasant stimuli, and with higher maximum responses (Table 2). Factor 2 (related to more behavioral aspects such as criminal behavior and irresponsibility) was found to be inversely correlated with instability of response to unpleasant stimuli: the higher the scores on this factor, the lower the response instability (Table 2).

**Table 2:** **Correlation indices (rho) between PCL-R scores and EDA measures for unpleasant stimuli**.

	Factor 1	Factor 2
EDA lat	0.5302*	−0.0105
EDA lab	−0.1369	−0.5743*
EDA max	0.5508*	−0.0901

EDA, electrodermal activity; lat, response latency; lab, response lability; max, peak of response; factors 1 and 2, PCL-R score in each factor. *rho index showing correlation with *p* < 0.005.

The results of the exploratory analysis of EDA correlations with pleasant and neutral stimuli are displayed in Table 3. The only significant finding consisted of a negative correlation between EDA liability during presentation of neutral images and factor 2 scores.

**Table 3:** **Correlation indices (rho) between PCL-R scores and EDA measures for pleasant and neutral stimuli**.

	Factor 1	Factor 2
EDA lat *p*	0.2065	−0.2592
EDA lat *n*	0.0105	−0.0617
EDA lab *p*	0.0830	−0.2312
EDA lab *n*	−0.1863	−0.5206*
EDA max *p*	0.0789	−0.2714
EDA max *n*	0.3050	0.1049

EDA, electrodermal activity; lat, response latency; lab, response lability; max, peak of response; factors 1 and 2, PCL-R score in each factor. *rho index showing correlation with *p* < 0.005.

### Correlation between EDA and recidivist behavior

None of the EDA parameters distinguished between the recidivist and non-recidivist groups (Table 4).

**Table 4:** **EDA measures in non-recidivist and recidivist juvenile offenders**.

Variable	Group	*M*	SD	Significance
EDA lat *p*	Recidivists	1.30	2.19	*p* = 0.25
	Non-recidivists	2.16	1.68	
EDA lat *u*	Recidivists	1.70	1.55	*p* = 0.88
	Non-recidivists	1.80	1.86	
EDA lat *n*	Recidivists	1.64	2.09	*p* = 0.28
	Non-recidivists	0.97	1.10	
EDA lab *p*	Recidivists	0.27	0.25	*p* = 0.11
	Non-recidivists	0.15	0.13	
EDA lab *u*	Recidivists	0.09	0.07	*p* = 0.30
	Non-recidivists	0.07	0.03	
EDA lab *n*	Recidivists	0.11	0.02	*p* = 0.16
	Non-recidivists	0.07	0.01	
EDA max *p*	Recidivists	52.50	361.75	*p* = 0.91
	Non-recidivists	42.31	80.30	
EDA max *u*	Recidivists	−10.86	152.53	*p* = 0.47
	Non-recidivists	20.52	67.22	
EDA max *n*	Recidivists	−40.70	204.87	*p* = 0.441
	Non-recidivists	0.09	54.37	

*EDA, electrodermal activity; lat, response latency; lab, response lability; max, peak of response; factors 1 and 2, PCL-R score in each factor*.

## Discussion

To the best of our knowledge, the present psychophysiological study is the first of its kind to assess young offenders taking into account gradient rates of psychopathy as assessed using the PCL-R. Our findings extended the results of previous studies in this field, as we detected significant correlations between specific aspects of EDA in response to the emotion-eliciting stimuli versus scores of psychopathy as assessed with the PCL-R.

Overall, relatively modest scores were found in the present sample for the two PCL-R factors, slightly lower than those reported in the international literature (32). Also notably, none of the subjects met formal criteria for psychopathy based on the PCL-R diagnostic threshold. Such pattern of PCL-R scores could be at least partially explained by the greater influence of social factors predisposing individuals to commit crimes in low-income environments compared to high-income settings, in which constitutional factors of the subjects tend to have a greater influence (33).

Nevertheless, despite the relatively modest PCL-R scores of our incarcerated sample, the fact that EDA measures were significantly correlated with scores on both factors 1 and 2 of the PCL-R indicates the validity of treating psychopathy as a continuous, dimensional variable in psychophysiological investigations. This provides reinforcement to the view that psychopathy should not be seen as a categorical diagnosis, but instead a dimensional construct whose biological correlates can be conceivably identified.

We found significant correlations between PCL-R scores and EDA indices only when the two factors of the scale were analyzed separately. This underscores the independence between the two PCL-R factors (4, 5, 10, 12), as well as the validity of investigating separately their neurobiological correlates. Moreover, we found that each characteristic of the electrodermal response of individuals to negative valence stimuli were distinctly related to scores on factors 1 or 2 of the PCL-R. This suggests that separate investigations of these two factors have the potential to unravel more complex relationships between EDA and the degree of psychopathy than simple, straightforward comparisons between subjects categorically diagnosed as psychopaths versus non-psychopaths.

The analyses for factor 1, which denotes emotional callousness and is more characteristic of psychopathy than factor 2 (34), revealed a positive correlation with latency time for physiological responses to pictures with negative emotional content. These findings are consistent with the prediction that higher scores on factor 1 of the PCL-R reflect empathic detachment, as indicated by the biological index of delayed EDA. These results extend those of previous investigations which reported temporal delays in reacting to stimuli in samples of psychopaths in a diversity of experimental designs, including measures of brain potential and startle reflex (35). Such pattern of response is thought to reflect a key impairment in the automatic recognition of affective contents in association with psychopathic traits (36).

However, despite such reasoning, yet another interpretation is possible, once offenders (specially violent ones) have difficulties in allocating attention to emotional stimuli (37). Criminals show impairments in shifting between perceptual dimensions (mostly due to deficits in inhibitory cognitive control), and moreover, they display an impaired ability to alter their behavior in response to fluctuations in the emotional significance of stimuli (37). Thus a lateness to engage in the stimuli content could be an alternative explanation for the increased latency of response in our subjects.

We also identified a significant positive correlation between factor 1 scores and peak EDA reaction. Thus a higher degree of psychopathy was related to a longer latency to react as discussed above, but also to more intense reactions to negative pictures. Although there is a tendency in the EDA literature to relate antisocial behavior with under-arousal [see Ref. (17), for a review], there are other studies showing that antisocial behavior may be even facilitated by increased arousal (20, 22). Comparing antisocial subjects with and without a history of emotional violence, Lakosina and Trunova (38) found larger EDA responses in the violent group (22). Other studies indicate a relationship between increased physiologic arousal, negative emotionality, and aggression/antisocial behavior (39–41), with a greater risk of aggression in subjects brought up in violent/stressful environments (42), as is the case of our subjects.

The behavioral expression of an enhanced degree of arousal will depend on the context, cognition, and learning, but the inability to regulate a negative affect and arousal is a major variable in the violent outcome of the emotions (22). Emotion regulation involves reducing autonomic arousal and focusing attention, using strategies such as distraction and concentration to goals when facing strong emotions (43, 44). It is noteworthy that mixed results regarding EDA and externalizing behavior problems have been found depending on the age of the subjects, as there is a period of transition in the psychophysiological response: children with both aggressive and delinquent behavior seem to have greater electrodermal responses, and throughout late childhood and adolescence this pattern undergoes gradual changes (45). In effect, elders have weaker responses to affective stimuli with negative content, probably because of the enhanced ability to control and sooth their reactions (46).

It is also important to bear in mind that non-empathic negative stimuli have been invariably used in the previous psychophysiological literature on antisocial and aggressive behavior to date, including aversive (e.g., pictures of mutilated bodies), anger-provoking (e.g., loud noises), or painful (e.g., electrical shocks) stimuli (17). Rather than that, we have employed a set of visual stimuli expected to produce empathic reactions, of either unpleasant or pleasant nature. There has been a lack of studies using empathic pictures in previous psychophysiological studies, despite the fact that lack of empathy is a cornerstone in the evaluation of psychopathy (47).

As far as we know, ours is the first EDA study of psychopathy to use images that trigger empathic responses; this could also help to explain the unexpected positive correlation of factor 1 scores and peak of response, given that psychopathy is characterized by fearlessness, but also by poor emotional control. EDA reflects general autonomic reactions, without identifying the specific emotion elicited in each subject by the stimuli; thus rather than fear, anger, or sadness, a higher EDA peak in individuals with greater scores on PCL-R factor 1 could actually reflect their stronger desire toward a stimulus of apparent negative emotional valence (48). Such pattern of reaction would not be expected to be elicited if we had chosen to use strictly aversive pictures of non-empathic content.

It has recently been proposed that externalizing behaviors, such as aggression and rule-breaking, are predicted by a specific pattern of psychophysiological reactions, which include low levels of inhibition and low sensitivity to punishment – the individuals with this profile would have difficulty in dealing with impulses and emotions, but would also be fearless, and thus late to learn from experience and punishment (49). Since we assess empathic emotions rather than responses to fear in the present study, our results may reflect this pattern.

In regard to factor 2 (corresponding to life style and behavior), we found that higher scores were significantly associated to lower EDA response lability, indicating that the higher the antisocial behavior indices, the lower the instability of electrodermal responses. This finding is consistent with the notion that lower autonomic control, while relating to a greater tendency for episodes of reactive aggressiveness, is also associated to a lower level of criminal behavior (50) – probably because intended crime requires a certain degree of coldness. A higher level of impulsivity, which has been associated with greater variability in EDA in previous studies (51), is most probably detrimental for planned criminal behavior. Our results extend those of previous studies which reported significant associations between behavioral aspects of psychopathy to lower levels of excitability (52).

Given that the PCL-R is a measure that essentially assesses the risk of criminal recidivism, we expected to find higher scores among recidivists compared to non-recidivist subjects. Additionally, in view of the significant correlations between PCL-R scores and EDA indices discussed above, we also believed that EDA patterns would be associated with criminal recidivism. In regard to the PCL-R, our results show that scores on this scale do differentiate young non-recidivist offenders from recidivist offenders. This result is unsurprising, since the scale is specifically designed to calculate risks of future criminal behavior. This relationship however, was not present for EDA parameters. We interpret this dissociation as indicative that emotional callousness alone is insufficient to explain criminal behavior. In contrast with the broad coverage of the PCL-R, EDA measurements alone are likely to be insufficient to address the multiplicity of factors linked to crime, and independently predict criminal recidivism. However, we must acknowledge that such relationship might have proved significant had we been able to recruit a larger sample. In a previous study involving 1795 individuals, Gao found an association between poor childhood fear conditioning (as measured by EDA) and criminal behavior 20 years later (52). Because the size of this effect was considerably modest (0.02 μS), a very large sample was demanded in order to afford sufficient power (52).

Other distinctive methodological aspect of the present study is that our subjects were confined for continued periods of time in a highly controlled setting, providing an environment that fulfilled the criteria of a “total institution,” defined by Goffman as “a place of residence and work where a large number of like-situated individuals, cut off from the wider society for an appreciable period of time, together lead an enclosed, formally administered round of life” (53). In the previous psychophysiological literature on psychopathy, there has been a high degree of variability in regard to methods for EDA measurements and characteristics of the samples investigated (54), making it difficult to avoid the influence of confounding variables which may affect EDA measurements.

For instance, the majority of studies conducted with incarcerated samples have pooled together, in the same experimental group: subjects with wide age ranges; subjects with positive or absent history of use alcohol and drugs; subjects who presented different types of psychiatric comorbidities; and subjects who had undergone widely variable periods of incarceration (5). All these are factors that may significantly influence EDA measurements (5, 55).

Other studies, comparing psychopaths and non-psychopaths using non-incarcerated samples, may have had their results confounded by inter-individual differences in other variables that also affect psychophysiological responses and which cannot be easily manipulated in non-controlled environments, such as dietary habits (56, 57) and variations in the levels of stress imposed to individuals in their daily living (58, 59).

Taken together, these limitations justify the view that “the literature on the psychophysiology of antisocial spectrum behavior is often confusing and seemingly contradictory” (17). Our study minimized the influence of such confounding variables, as it focused on a population of incarcerated youngsters from a single institution, homogeneously distributed in regard to gender (male), age range and level of education, and with no major psychiatric comorbidities. By assessing individuals after a minimum period of 3 months of incarceration, we excluded confounding influences such as dietary variations and recent use of alcohol and other drugs of abuse.

One other relevant methodological aspect of the present study was the absence of a control group to enable comparison against the study subjects. The methodology elected, however, was specifically chosen with the intention of assessing a homogeneous population, with all individuals drawn from the same setting and submitted to the same daily regimen of influences. If we had sought to compare the psychophysiological response of young offenders against non-offenders, the confounding issues would have rendered the election of a control group logistically unattainable, since it would not be possible to find a sufficient number of individuals who would be non-offenders but yet displaying similar traits as the young offenders. In the present study, a large number of factors influencing personality, psychophysiological response as well as criminal behavior were controlled for, allowing differences to be ascribed predominantly to the factors analyzed.

In conclusion, by investigating a population that was homogenous in terms of gender, age, background, and exposure to external influences, our results reinforce the association between the degree of psychopathy (as a continuous, quantifiable trait) and psychophysiological responses. These results extend previous literature findings by attesting that there is a negative correlation between latency of psychophysiological response and psychopathy, even when this is assessed in a dimensional fashion. Also, by demonstrating such association using visual stimuli showing empathic content, our results provide a link between lack of empathy (as assessed by PCL-R scores) and a biological index of empathic detachment.

Finally, the pattern of correlations between PCL-R scores and EDA identified herein may indicate that, in part, the emotional callousness phenotypically expressed by young offenders may be an environmentally prompted behavior, more than being physiologically determined. Further studies employing this experimental design are warranted involving larger samples and controlling for the influence of other variables such as type of offense perpetrated, an aspect that was beyond the scope of the present investigation.

## Conflict of Interest Statement

The authors declare that the research was conducted in the absence of any commercial or financial relationships that could be construed as a potential conflict of interest.
